# The Antifungal Mechanism of Isoxanthohumol from *Humulus lupulus* Linn.

**DOI:** 10.3390/ijms221910853

**Published:** 2021-10-07

**Authors:** Yin-Fang Yan, Tian-Lin Wu, Sha-Sha Du, Zheng-Rong Wu, Yong-Mei Hu, Zhi-Jun Zhang, Wen-Bin Zhao, Cheng-Jie Yang, Ying-Qian Liu

**Affiliations:** 1 School of Pharmacy, Lanzhou University, Lanzhou 730000, China; yanyf18@lzu.edu.cn (Y.-F.Y.); wutl19@lzu.edu.cn (T.-L.W.); dushsh19@lzu.edu.cn (S.-S.D.); wuzhengrong@lzu.edu.cn (Z.-R.W.); huym20@lzu.edu.cn (Y.-M.H.); zhaowb19@lzu.edu.cn (W.-B.Z.); yangchj16@lzu.edu.cn (C.-J.Y.); 2 State Key Laboratory of Grassland Agro-ecosystems, Lanzhou University, Lanzhou 730000, China

**Keywords:** *Humulus lupulus* Linn., isoxanthohumol, antifungal activity, *Botrytis cinerea*

## Abstract

*Humulus lupulus* Linn. is a traditional medicinal and edible plant with several biological properties. The aims of this work were: (1) to evaluate the in vitro antifungal activity of *H. lupulus* ethanolic extract; (2) to study the in vitro and in vivo antifungal activity of isoxanthohumol, an isoprene flavonoid from *H. lupulus*, against *Botrytis cinerea*; and (3) to explore the antifungal mechanism of isoxanthohumol on *B. cinerea*. The present data revealed that the ethanolic extract of *H. lupulus* exhibited moderate antifungal activity against the five tested phytopathogenic fungi in vitro, and isoxanthohumol showed highly significant antifungal activity against *B. cinerea*, with an EC_50_ value of 4.32 µg/mL. Meanwhile, it exhibited moderate to excellent protective and curative efficacies in vivo. The results of morphologic observation, RNA-seq, and physiological indicators revealed that the antifungal mechanism of isoxanthohumol is mainly related to metabolism; it affected the carbohydrate metabolic process, destroyed the tricarboxylic acid (TCA) cycle, and hindered the generation of ATP by inhibiting respiration. Further studies indicated that isoxanthohumol caused membrane lipid peroxidation, thus accelerating the death of *B. cinerea*. This study demonstrates that isoxanthohumol can be used as a potential botanical fungicide for the management of phytopathogenic fungi.

## 1. Introduction

Over decades, diseases and damages caused by phytopathogenic fungi can greatly diminish the yield and quality of crops [1]. It is estimated that the losses caused by phytopathogenic fungi total more than $200 billion each year, which is significantly higher than the losses caused by any other group of microorganisms [2]. Although chemical fungicides are still widely used to reduce plant diseases in agriculture, the abuse of these chemicals has led to toxic residues and resistance among plant pathogenic fungi, which has caused several environmental and health problems [3,4]. Given these major drawbacks and the fact that the public are increasingly sensitive to the use of all chemical fungicides, demand for novel, effective, and environmentally acceptable fungicides has been soaring rapidly [5].

In recent years, natural products have been considered an important and promising source for the research and development of new drugs due to their easy biodegradation and low residual toxicity [6]. Many natural products have been reported to be effective against phytopathogenic fungi [7]. For example, tephroapollin-F, a prenylated flavonoid isolated from *Tephrosia apollinea* L., effectively inhibited the growth of *Colletotrichum acutatum* [8]. Pinostrobin and flavokawin B are bioactive compounds from *Polygonum stelligerum* that have been demonstrated to effectively suppress the growth of *Monilinia fructicola* and *Rhizopus stolonifer* [9]. Furthermore, antidesmone, isolated from *Waltheria indica*, displayed broad-spectrum antifungal activities against all tested phytopathogenic fungi with EC_50_ values ranging from 0.60 to 20.15 μg/mL [1].

*Humulus lupulus* Linn. (Cannabaceae) is a traditional medicinal and edible plant, commonly named hops, that is widely distributed all over the world [10]. Because of the bitterness and the antiseptic and aromatic properties of its metabolites, it is particularly popular with brewers [11]. However, *H. lupulus* was originally used for medicinal purposes [12], and it has been demonstrated to display a variety of pharmacological properties, such as antioxidant, estrogenic, antimicrobial, and anti-inflammatory proprieties [13]. *H. lupulus* contains various terpenes, phytoestrogens, tannins, essential oils, and flavonoids, with abundant activity in vitro. There are many reports about the biological activities of flavonoids in *H. lupulus*. Mizobuchi reported that xanthohumol and 6-isopentenylnaringenin can effectively inhibit the growth of *Staphylococcus aureus*, with an MIC value of 6.25 μg/mL, while that of isoxanthohumol is 50.0 μg/mL [14]. Bocquet et al. demonstrated that desmethylxanthohumol in *H. lupulus* showed effective antifungal activity against *Zymoseptoria tritici* with an MIC value of 0.63 g/L [15]. At the same time, Agnieszka et al. also found that xanthohumol can effectively inhibit the growth of three *Fusarium* species, with MIC_50_ values ranging from 0.015 to 0.100 mg/mL [16]. To the best of our knowledge, isoxanthohumol found in *H. lupulus* has not been reported for potential antifungal activities against phytopathogenic fungi.

**Figure 1:**
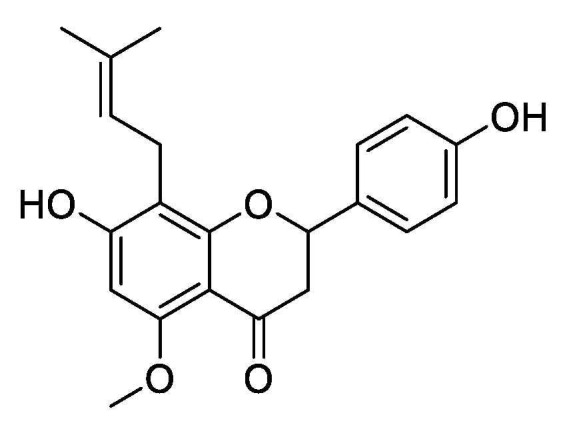
The structure of isoxanthohumol.

Thus, the present study aimed to quantify the content of isoxanthohumol (Figure 1) in *H. lupulus* and assess its in vitro and in vivo antifungal activity. Then, SEM and TEM were used to observe the effects of isoxanthohumol on *B. cinerea*, and transcriptomics technology was used to explore the antifungal mechanism of isoxanthohumol against *B. cinerea*. Finally, RT-qPCR and physiological indicators, such as total carbohydrate content, dehydrogenase activities, cell respiration, adenosine triphosphate (ATP) content, and ATPase activity, were used to validate the results of RNA-seq. Furthermore, we evaluated the effects of isoxanthohumol on *B. cinerea* by observing the changes in cell membrane permeability; the contents of glycerol, MDA, and H_2_O_2_; and antioxidant-related enzyme activity, which provide a theoretical basis for the development of isoxanthohumol.

## 2. Results and Discussion

### 2.1. Antifungal Activity of H. lupulus Extract and Its Isoxanthohumol Content

**Figure 2:**
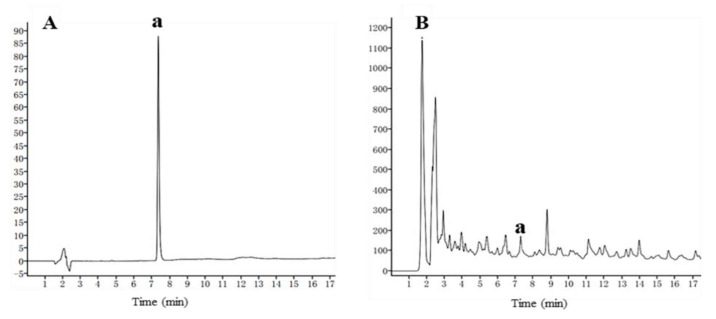
HPLC chromatograms of standards (**A**) and raw material (**B**); a: isoxanthohumol.

In this study, ethanolic extract of *H. lupulus* and isoxanthohumol were prepared firstly. Subsequently, their antifungal activity was determined in vitro. As shown in Table 1, ethanolic extract of *H. lupulus* exhibited moderate antifungal activity against all five pathogenic fungi (inhibition rate: 37.01~51.52% at 500 μg/mL). At the same time, isoxanthohumol, the main isoprene flavonoid from *H. lupulus*, showed more excellent antifungal activity against pathogenic fungi, especially *B. cinerea*. Then, the content of isoxanthohumol in *H. lupulus* was analyzed by HPLC, and the results showed that the content of isoxanthohumol is low (0.052 mg/g) (Figure 2).

### 2.2. Antifungal Activity of Isoxanthohumol

The antifungal activity of isoxanthohumol against three common pathogenic fungi in vitro is shown in Table 2: it was effective against *S. sclerotiorum*, *B. cinerea*, and *F. graminearum* with EC_50_ values of 14.52, 4.32, and 16.50 µg/mL, respectively. The inhibition rates at 50, 25, 10, 5, and 2.5 µg/mL are shown in Table S1. *B. cinerea* is a ubiquitous pathogenic fungus that can infect a wide range of commercial crops and cause important economic losses [17]. The mechanism of isoxanthohumol on *B. cinerea* remains largely unclear, so it is important to determine the mechanism of action of isoxanthohumol against *B. cinerea*.

**Figure 3:**
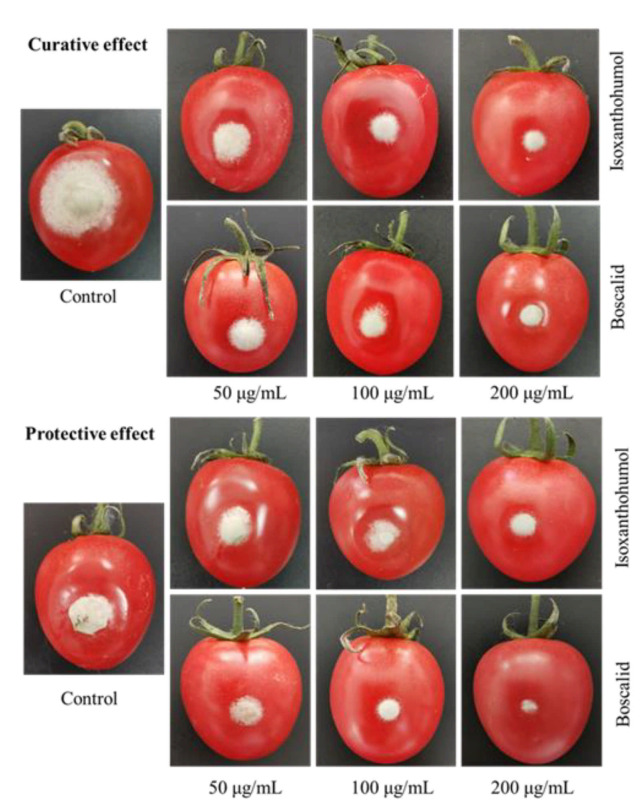
The antifungal activity of isoxanthohumol and boscalid in vivo.

The investigation into the in vivo antifungal activity showed that isoxanthohumol was effective at inhibiting the growth of *B. cinerea* in isolated cherry tomatoes. As we can see from Table 3 and Figure 3, *B. cinerea* infection caused serious tissue rot in the blank control. After treatment with 50 μg/mL, 100 μg/mL, and 200 μg/mL of isoxanthohumol for 5 days, the control efficacies were 27.39%, 35.23%, and 39.25%, respectively, in terms of curative effect. The protective efficacies of isoxanthohumol and boscalid (positive control) were 32.35%, 38.32%, 54.85% and 42.93%, 49.09%, 69.95%, respectively, at 50 μg/mL, 100 μg/mL, and 200 μg/mL. This study demonstrated moderate to excellent protective and curative effects of isoxanthohumol in vivo. Moreover, isoxanthohumol is the main isoprene flavonoid from the medicinal and edible plant *H. lupulus* [18], so it has the potential to be developed as a botanical fungicide to prevent plant diseases caused by *B. cinerea*.

### 2.3. Effect of Isoxanthohumol on B. cinerea Spore Germination

**Figure 4:**
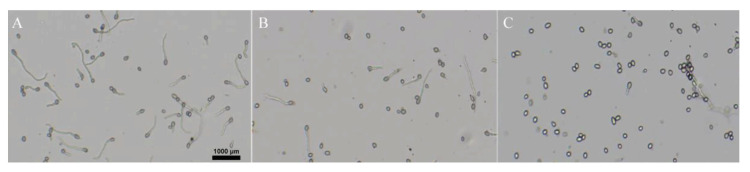
Inhibitory activity of isoxanthohumol on spore germination of *B. cinerea*: (**A**) Blank control, (**B**) 10 μg/mL, (**C**) 25 μg/mL.

Spores are known as the main source of infection for gray mold; once the environmental conditions are suitable, they germinate to infect the host plant [19]. The effect of isoxanthohumol on *B. cinerea* spore germination is shown in Figure 4. Most of the spores germinated in the blank control when cultured for 8 h, but the inhibition rate of spore germination reached 100% when treated with 25 μg/mL of isoxanthohumol. In conclusion, the spore germination of *B. cinerea* was inhibited in a dose-dependent manner.

### 2.4. Effect of Isoxanthohumol on B. cinerea Morphology

**Figure 5:**
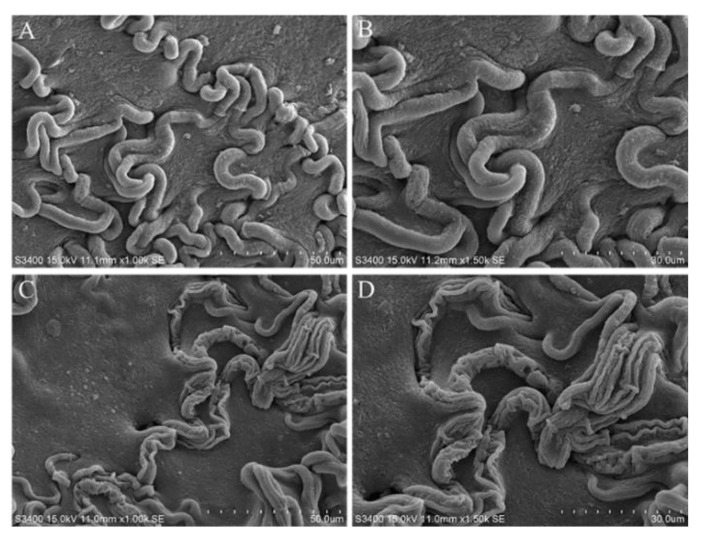
Scanning electron microscopy observations of the morphology of *B. cinerea* mycelia: (**A**) Blank control, 0.5% DMSO, ×10; (**B**) Blank control, 0.5% DMSO, ×1500; (**C**) Treated with 0.5% DMSO plus isoxanthohumol at 5 µg/mL, ×10; (**D**) Treated with 0.5% DMSO plus isoxanthohumol at 5 µg/mL, ×1500.

**Figure 6:**
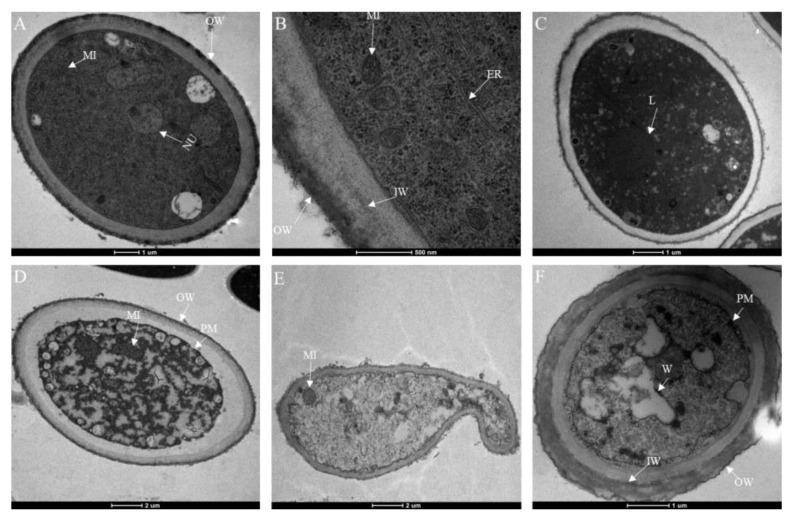
Transmission electron micrographs of the *B. cinerea* cell structure: (**A**–**C**) Blank control, 0.5% DMSO; (**D**–**F**) Treated with 0.5% DMSO plus isoxanthohumol at 5 µg/mL; OW: outer wall; IW: inner wall; MI: mitochondria; NU: nucleus; ER: endoplasmic reticulum; L: lipid body; PM: plasma membrane.

To examine the effect of isoxanthohumol on the morphology and ultrastructure of *B. cinerea*, SEM and TEM techniques were used. SEM analysis showed that isoxanthohumol caused considerable morphological alterations in *B. cinerea*. The mycelia in the blank control showed a uniform intact, homogenous, and robust morphology, and they exhibited smooth surfaces. By contrast, mycelia were deformed, bent, and collapsed after treatment with isoxanthohumol (Figure 5). The results of TEM observation indicated that isoxanthohumol could lead to significant alteration and disorganization of the cell ultrastructure. Compared to the blank control sample, *B. cinerea* under 5 μg/mL of isoxanthohumol displayed an abnormal cell ultrastructure, which caused lysis; the plasma membrane was thickened and damaged; mitochondria had slightly swollen; and plasmolysis was obvious. Some organelles, such as the liposome and nucleus, disappeared (Figure 6). All of these results demonstrated that isoxanthohumol caused significant damage to the morphology and ultrastructure of *B. cinerea*.

### 2.5. Transcriptome Analysis of B. cinerea under Isoxanthohumol Treatment

**Figure 7:**
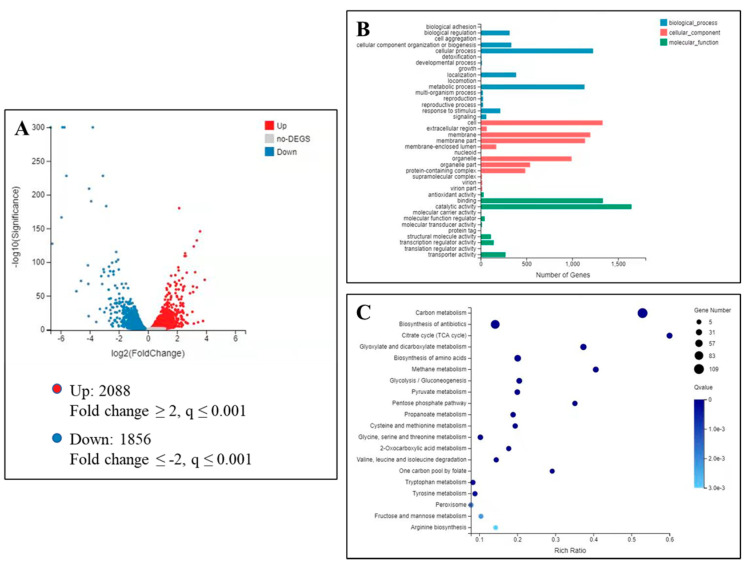
Transcriptome analysis of the effects of isoxanthohumol on *B. cinerea.* (**A**) A volcano plot of the DEGs from control/isoxanthohumol (the *X*-axis represents value A (log_2_-transformed mean expression level), the *Y*-axis represents value M (log_2_-transformed fold change)); (**B**) DEG numbers of the most enriched GO terms; (**C**) KEGG enrichment analysis of DEGs.

To better understand the molecular changes underlying the impact of isoxanthohumol on *B. cinerea*, a transcriptome profiling assay was performed on the two samples—a blank control and treatment (5 μg/mL of isoxanthohumol). Transcriptome analysis of the effects of isoxanthohumol on *B. cinerea* is showen in Figure 7. As illustrated in the volcano plot (Figure 7A), 3944 significantly differentially expressed genes (DEGs) were screened, among which 2088 DEGs were significantly upregulated and 1856 were downregulated in the treatment group when compared with the blank control (|log_2_(Fold Change)| ≥ 2 and *q* value ≤ 0.001). Subsequently, the significantly differentially expressed genes (DEGs) were compared with the Gene Ontology (GO) database, and the DEGs were found to be mainly involved in three types of processes: biological process, cellular component, and molecular function (Figure 7B). Next, 334 DEGs related to the metabolic process in the biological process were chosen to execute GO enrichment analysis and Kyoto Encyclopedia of Genes and Genomes (KEGG) enrichment analysis, and the results indicated the expression of 109 DEGs involved in the carbon metabolism pathway and another 27 DEGs involved in the TCA cycle pathway (Figure 7C). Further analysis demonstrated that these DEGs mainly focus on the ATP metabolic process, generation of precursor metabolites and energy, and purine ribonucleotide metabolic process, especially energy metabolism, carbohydrate metabolism, cell growth, and amino acid metabolism. Key enzyme genes associated with the TCA cycle, including citrate synthase, succinate dehydrogenase, and other dehydrogenases, were also differentially expressed. Therefore, we speculate that isoxanthohumol may cause significant damage to the whole metabolic pathway, including carbon metabolism, the TCA cycle, ATP generation, and respiration. Based on this speculation, we evaluated the effects of isoxanthohumol on the total carbohydrate content, dehydrogenase activities, CA content, ATP content, ATPase activity, and cell respiration to validate the results of transcriptomics.

### 2.6. Verification of DEGs by RT-qPCR

**Figure 8:**
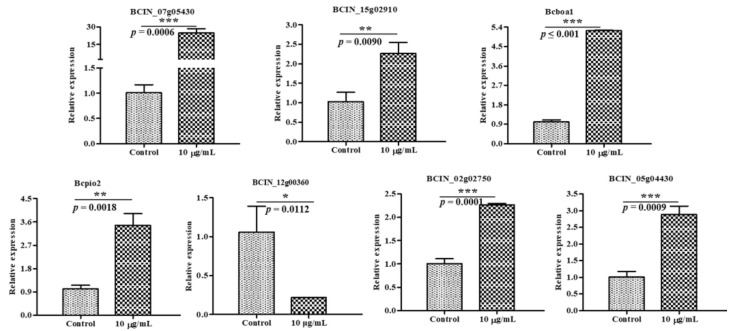
Verification of DEGs by RT-qPCR. * *p* < 0.05, ** *p* < 0.01 and *** *p* < 0.001.

To validate the expression of the DEGs identified in the transcriptome results for the *B. cinerea* mycelia, seven genes (six upregulated genes and one downregulated gene) were randomly selected from the results for RT-qPCR analysis. Within the seven selected genes, five genes were related to carbon metabolism and two genes were related to the TCA cycle. It can be seen in Figure 8 that all the changes in the chosen genes tested in RT-qPCR were consistent with the results of the RNA-Seq, and some genes were found to be most significantly different in the blank control and treatment, which confirmed that the RNA-seq data were reliable.

### 2.7. Effects of Isoxanthohumol on B. cinerea Total Carbohydrate Content and TCA Cycle

**Figure 9:**
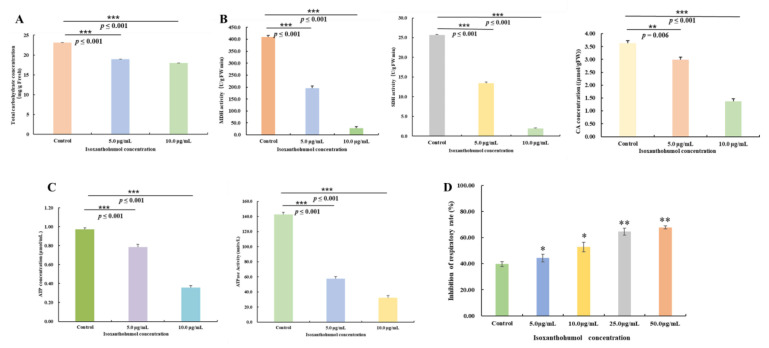
(**A**) Effect of isoxanthohumol on the total carbohydrate content of *B. cinerea*; (**B**) Effects of isoxanthohumol on the dehydrogenase activities and CA content of *B. cinerea*; (**C**) Effects of isoxanthohumol on the ATP content and ATPase activity of *B. cinerea*; (**D**) Inhibition rate of isoxanthohumol on cell respiration of *B. cinerea* mycelia. * *p* < 0.05, ** *p* < 0.01 and *** *p* < 0.001.

The DEGs involved in the carbohydrate metabolic process were found to be most significantly different among the transcriptome results. Carbohydrates are the main carbon sources in the TCA cycle, and inhibition of them would cause damage to the metabolic pathways. Carbohydrate metabolism is one of the main metabolic pathways and forms of energy supply in fungi, including biochemical pathways for biosynthesis, degradation, interconversion, and energy metabolism [20,21]. Effect of isoxanthohumol on the mycelial metabolism was evaluated (Figure 9). Figure 9A shows that isoxanthohumol decreased the total carbohydrate content of *B. cinerea* in a concentration-dependent manner. After treatment with 5.0 μg/mL and 10.0 μg/mL of isoxanthohumol, the total carbohydrate content was decreased by 18.23% and 22.41%, respectively. This indicated that the carbohydrate metabolic capability of *B. cinerea* was seriously impaired by isoxanthohumol.

The DEGs involved in the TCA cycle were also noticed by their amount and the significance of the transcriptome results. Carbohydrates are the main carbon sources in the TCA cycle, and inhibition of them would cause damage to the TCA cycle [22]. MDH plays an important role in the energy-producing pathway and catalyzes the interconversion of malate to oxaloacetate, while SDH catalyzes the oxidation of succinate to fumarate in the TCA cycle [23]. The activities of key enzymes related to the TCA cycle are shown in Figure 9B. Compared with the blank control, the SDH and MDH enzyme activities demonstrated most significant decreases (*p* < 0.001) in *B. cinerea* cells as the concentration of isoxanthohumol increased. The MDH activities were decreased by 52.36% and 93.19% at 5.0 μg/mL and 10.0 μg/mL of isoxanthohumol, respectively, and the SDH activities were decreased by 47.50% and 92.50%, respectively, at the same concentrations. Intracellular citrate functions as a key regulator of energy metabolism, modulates ATP production, and is the prime carbon source for fatty acid synthesis in the cytoplasm [24]. The CA content in *B. cinerea* was also inhibited by isoxanthohumol at concentrations of 5.0 and 10.0 μg/mL, decreasing by 17.78% and 62.39%, respectively, compared with the blank control. This demonstrated that isoxanthohumol may inhibit the growth of *B. cinerea* by affecting the TCA cycle.

Considering that the intracellular ATP content directly affects the normal energy metabolism of pathogenic fungi, is a general energy source for all living cells, and provides direct energy for various life activities of organisms [25], the effects of isoxanthohumol on ATP content and ATPase activity were also studied. As shown in Figure 9C, the ATP content in *B. cinerea* was 0.9737 μmol/mL in the blank control; when treated with 5.0 and 10.0 μg/mL of isoxanthohumol, the ATP content reduced to 0.7859 μmol/mL and 0.3590 μmol/mL, respectively, and ATPase activity was decreased by 59.74% and 77.29%, respectively, at the same concentrations.

In general, the respiration of pathogenic fungi functions by absorbing oxygen to release energy, some of which is stored as ATP. In addition, respiratory metabolism can improve the vitality of pathogenic fungi [17]. The effect of isoxanthohumol on cell respiration is shown in Figure 9D. The isoxanthohumol decreased the respiration of *B. cinerea* in a concentration-dependent manner. After treatment with isoxanthohumol for 24 h at concentrations of 5.0, 10.0, 25.0, and 50.0 μg/mL, the inhibition rates of respiration were 44.31%, 52.78%, 64.47%, and 67.75%, respectively. This indicated that the respiration of *B. cinerea* was significantly inhibited by isoxanthohumol compared with the blank control (*p* < 0.05). All of these results together demonstrate that isoxanthohumol affects the carbohydrate metabolic process, destroys the TCA cycle, and hinders the generation of ATP by inhibiting respiration, thus accelerating the death of *B. cinerea*.

### 2.8. Changes Due to Isoxanthohumol in B. cinerea Cell Membrane

**Figure 10:**
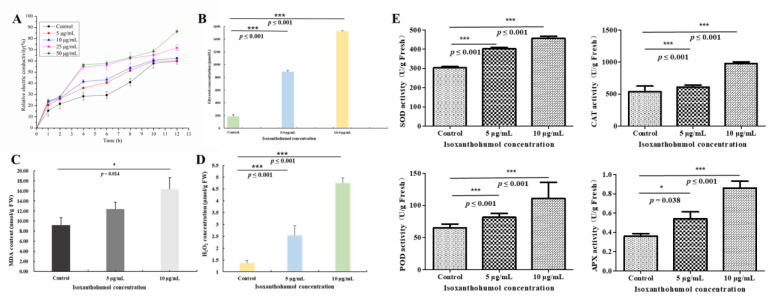
(**A**) Effect of isoxanthohumol on the cell membrane permeability of *B. cinerea* mycelia; (**B**) Effect of isoxanthohumol on the glycerol content of *B. cinerea*; (**C**) Effect of isoxanthohumol on the MDA content of *B. cinerea* (**D**) Effect of isoxanthohumol on the H_2_O_2_ content of *B. cinerea*; (**E**) Effect of isoxanthohumol on the activity of antioxidant-related enzymes of *B. cinerea*. * *p* < 0.05 and *** *p* < 0.001.

The relative electric conductivity of the culture medium is used to reflect the cell membrane permeability of mycelia [26], and the permeability and fluidity of the cell membrane are of considerable significance to the survival of cells. Previous research demonstrated that flavonoids exert their antifungal activity by destroying the cytoplasmic membrane [27], and xanthohumol could also increase membrane permeability in HL-60 cells [28]. Isoxanthohumol is the main isoprene flavonoid in *H. lupulus*, and the morphology results in our study also showed that isoxanthohumol caused obvious damage to the cell membrane of *B. cinerea* (Figure 10). Therefore, we speculate that isoxanthohumol may also exert antifungal activity by destroying the cell membrane. Figure 10A shows that the relative electrical conductivity was increased in a dose-dependent and time-dependent manner. Although the discrepancy in the relative electric conductivity was not significant in the initial 4 h, the relative electric conductivity in the treatment with 50.0 μg/mL of isoxanthohumol was significantly higher than those for all of the other, lower concentrations in the subsequent time period.

The cell membrane is essential for the normal growth of cells, and destruction of the cell membrane causes high osmotic pressure [29]. It has been demonstrated that intracellular glycerol plays an important role in the response to osmotic stress, and the process of osmotic adaptation by activation of the HOG pathway results in the biosynthesis and accumulation of glycerol to balance the osmotic pressure [30]. To further verify whether isoxanthohumol affected osmotic stress, we tested the change in glycerol content. As shown in Figure 10B, the intracellular glycerol content increased most significantly in isoxanthohumol-treated *B. cinerea* compared with the blank control (*p* < 0.001). This indicated that isoxanthohumol induced cell membrane damage in *B. cinerea*.

### 2.9. Effect of Isoxanthohumol on Membrane Lipid Peroxidation of B. cinerea

MDA is one of the main indexes of lipid peroxidation, and its content is usually used as an indicator of oxidative stress. The formation of MDA results in the loss of cell membrane integrity and damage to pathogenic fungi [31]. As shown in Figure 10C, the MDA content in *B. cinerea* increased significantly after treatment with 5 and 10 μg/mL of isoxanthohumol (*p* < 0.05). The membrane lipid peroxidation of *B. cinerea* mycelia in this study may be related to the changes in MDA content.

H_2_O_2_ is a kind of reactive oxygen species; it is effective at eliciting a signaling response to enhance the tolerance and defense ability of mycelia, but it may cause oxidative damage to lipids, proteins, and nucleic acid molecules [32]. As illustrated in Figure 10D, the H_2_O_2_ content of *B. cinerea* mycelia was increased in a dose-dependent manner. After treatment with 5.0 μg/mL and 10.0 μg/mL of isoxanthohumol, H_2_O_2_ contents were increased by 46.40% and 71.20%, respectively. This result indicates that the membrane lipid peroxidation of *B. cinerea* mycelia may be associated with an increase in H_2_O_2_ content.

The H_2_O_2_ content changes with the activity of antioxidant enzymes, including SOD, CAT, POD, and APX enzymes [4], and the antioxidant enzymes can be used to reflect whether mycelia are damaged by membrane lipid peroxidation [31]. As shown in Figure 10E, antioxidant-related enzymes of *B. cinerea* mycelia were increased in a dose-dependent manner, and the most significant difference in SOD, CAT, POD, and APX activity was observed between the isoxanthohumol treatment and blank control (*p* < 0.001). All of these results demonstrated that isoxanthohumol may cause the destruction of the cell membrane by membrane lipid peroxidation, which causes final damage to *B. cinerea* mycelia.

## 3. Materials and Methods

### 3.1. Pathogenic Fungi and Reagents

Isoxanthohumol (CAS-No. 70872-29-6; 98%) was purchased from Sichuan Weikeqi Biological Technology Co., Ltd. (Chengdu, China). The Fungal RNA kit was purchased from Beijing Noble Technology Co., Ltd. (Beijing, China). The one-step RT-PCR kit and SuperReal PreMix (SYBR Green) were purchased from Tiangen Biotech (Beijing, China) Co., Ltd. The yeast mitochondria isolation kit and ATP colorimetric/fluorometric assay kit were purchased from Biovision (San Francisco, CA, USA). The ATPase/GTPase activity assay kit was purchased from Sigma-Aldrich (St. Louis, MO, USA). The BCA protein assay kit, NAD-malate dehydrogenase (NAD-MDH) assay kit, micro succinate dehydrogenase (SDH) assay kit, citric acid (CA) content assay kit, total carbohydrate colorimetric assay kit, micro malondialdehyde (MDA) assay kit, micro hydrogen peroxide (H_2_O_2_) assay kit, superoxide dismutase (SOD) assay kit, micro catalase (CAT) assay kit, micro peroxidase (POD) assay kit, and micro ascorbate peroxidase (APX) assay kit were purchased from Solarbio (Beijing, China). The glycerol assay kit was purchased from Nanjing Jiancheng Bioengineering Institute (Nanjing, China). *H. lupulus* Linn. was provided by Shaanxi Fangsheng Medical Technology Co., Ltd. (Hanzhong, China) and was identified by Prof. Yang Zhigang. The voucher specimen was submitted to the herbarium of the Lanzhou Institute of Pharmacology (Lanzhou University, China).

Five pathogenic fungus isolations—*Rhizoctonia solani*, *Sclerotinia sclerotiorum*, *Botrytis cinerea*, *Fusarium graminearum*, and *Magnaporthe oryzae*—were provided by the Institute of Plant Protection, Gansu Academy of Agricultural Science. The fungi were cultured in potato dextrose agar medium (PDA) at 23 ± 2 °C for a week to obtain new mycelia for the antifungal assay.

### 3.2. H. lupulus Extracts and HPLC Analysis

The powdered material of *H. lupulus* (40.00 g) was ultrasonically extracted three times with 80% ethanol (480 mL × 3.25 h × 3) at 65 °C. The ethanolic extract was combined, filtered, and concentrated at 45 °C under reduced pressure [33]. The extract was obtained after evaporation.

The sample was filtered using a nylon membrane (0.22 µm pore size). Then, 1.0 mL of the standard and extract were placed in vials for analysis by HPLC at 30 °C with a UV–vis photodiode array detector at 288 nm (1260 Infinity II, Agilent, Santa Clara, CA, USA). Determination was performed under the following operating conditions: a reversed-phase column (C18, 4.6 mm × 150 mm; particle size 5 μm, Agilent, Santa Clara, CA, USA, ZORBAX SB-C18); mobile phase of acetonitrile/1% formic acid solution in gradient elution (0~20 min: 40–60, 20~25 min: 100–0, 25~28 min: 40–60, 28~33 min: 40–60, *v*/*v*) at a flow rate of 0.8 mL/min; and an injection volume of 5 µL. Finally, the content of isoxanthohumol in *H. lupulus* was calculated based on the obtained calibration curve.

### 3.3. In Vitro Antifungal Activity

The mycelium growth inhibition method was used to evaluate the in vitro antifungal activity of the ethanolic extract of *H. lupulus* and isoxanthohumol. In this method, extract and isoxanthohumol were dissolved in dimethyl sulfoxide (DMSO) and then added to PDA medium to obtain sterile media of different concentrations. Boscalid and 0.5% DMSO (*v*/*v*) were used as a positive control and blank control, respectively. A 5 mm agar plug of each fungal strain, obtained from a 3-day-old PDA culture, was inoculated in the middle of PDA plates containing DMSO or samples. Petri dishes were incubated at 23 ± 2 °C in darkness, and radial growth was recorded by measuring the cross diameters of three replicates. The antifungal activity in vitro was expressed as the EC_50_ value, and the mycelial growth inhibition rate was calculated by the following formula:

Mycelial growth inhibition (%) = [(dc − dt)/(dc − 5 mm)] × 100

where dc and dt are the average diameters of the control and treatment fungal colonies, respectively.

### 3.4. In Vivo Antifungal Activity

To prepare test solutions at concentrations of 200 μg/mL, 100 μg/mL, and 50 μg/mL, isoxanthohumol was dissolved in DMSO, followed by dilution with water containing Tween 20 (Solarbio, Beijing, China). Next, cherry tomatoes all similar in size and growth status and without infection by pathogens were selected and washed with clean water, surface sterilized with 2% NaOCl for 5 min, and rinsed three times in sterile water. A 3 mm deep hole was pricked on the skin of each cherry tomato, and a drop of sample solution (10 μL) was injected into it. Boscalid (Solarbio, Beijing, China) and 0.5% DMSO (*v*/*v*) were used as a positive control and blank control, respectively. Twenty-four hours later, 5 μL of conidia (1 × 10^5^ ~ 1 × 10^6^ spores/mL) was inoculated. After that, cherry tomatoes were placed in an artificial climate chamber at 23 ± 2 ℃ and 85% relative humidity for 7 days (Tao et al., 2020). For curative efficacy, 5 μL of conidia was inoculated before isoxanthohumol solution was injected. All experiments for in vivo antifungal activity were run in triplicate, and the protective efficacy or curative efficacy was calculated.

### 3.5. Effect of Isoxanthohumol on B. cinerea Spore Germination

A spore suspension of *B. cinerea* was obtained from 10-day-old cultures and mixed with sterile distilled water to obtain a homogeneous spore suspension of 1 × 10^6^ spores/mL. A 100 μL spore suspension of *B. cinerea* was placed on a depression slide containing 100 μL of different concentrations of isoxanthohumol and added 1% DMSO, with sterile distilled water as a blank control. The inoculated glass slides were incubated in a digital biochemical incubator at 23 ± 2 ℃. Spore germination was observed under the microscope at 8 h after inoculation [3]. Each experiment was performed three times, and the inhibition rate of spore germination was calculated.

### 3.6. Scanning Electron Microscopy (SEM) Observations

The effect of isoxanthohumol on the morphological characteristics of *B. cinerea* was observed using a scanning electron microscope (SEM). A sample was obtained from the colonies of 3-day-old cultures and fixed with 2.5% glutaraldehyde for 4 h, then washed three times with 0.1 M phosphate buffer (pH 7.2) for 10 min each time. The sample was post-fixed with 1% osmium tetraoxide solution for another 2 h, then dehydrated in an ethanol gradient of 20%, 50%, 80%, and 100% for 10~15 min each. Finally, the sample was critical point dried and then coated with gold. It was visualized under a scanning electron microscope (Hitachi, S-3400N, Tokyo, Japan) at an accelerating voltage of 15.0 kV.

### 3.7. Transmission Electron Microscopy (TEM) Observations

The mycelium was prepared, as mentioned above, for examination under transmission electron microscope (TEM). The sample was pre-fixed in 2.5% glutaraldehyde in 0.1 M phosphate buffer (pH 7.2) for 4 h at 23 ± 2 ℃, and then washed three times in 0.1 M phosphate buffer for 10 min each. The mycelium was post-fixed in 1% osmium tetroxide for another 2 h and dehydrated in a graded series of ethanol and acetone. Finally, it was embedded in Epon 812 (Sigma Aldrich, St. Louis, MO, USA) and polymerized in Spurr’s resin at 60 °C for 48 h. The specimen block was sectioned to 70 nm thickness, then double-stained with uranyl acetate and lead citrate. The stained section was examined via TEM (JEM-1010 transmission electron microscopy; NEC, Tokyo, Japan).

### 3.8. Transcriptome Analysis

The *B. cinerea* mycelia used in this experiment were obtained from cultures on PD medium for 3 d. An agar plug of *B. cinerea* strain from a 3-day-old PDA culture was added to PD medium containing 5 μg/mL of isoxanthohumol used as treatment, and 0.5% DMSO (*v*/*v*) was used as a blank control simultaneously. Each group contained three biological replicates, and the harvested mycelia were quickly frozen in liquid nitrogen. The total RNA extraction, quantification, qualification, cDNA library construction, and transcriptome sequencing were completed using a service from Beijing Genomics Institute (BGI) Co., Ltd. (Shenzhen, China). The sample was sequenced on the BGIseq500 platform (BGI-Shenzhen, China).

### 3.9. Verification of DEGs by RT-qPCR

Seven random DEGs related to carbon metabolism or the citrate cycle (TCA cycle) were validated by RT-qPCR with three replicates to verify the transcriptome data. The total RNA extracted was determined as described using RNA isolation solvent (Omega Bio-tek, Norcross, GA, USA). For the reverse transcription reaction, cDNA was synthesized from 500 ng of total RNA using a FastKing gDNA Dispelling RT SuperMix kit (Tiangen Biotech Co., Ltd., Beijing, China) according to the manufacturer’s instructions. Real-time fluorescence quantitative PCR (qPCR) was performed using a SuperReal PreMix Plus (SYBR Green) kit (Tiangen Biotech Co., Ltd., Beijing, China) according to the manufacturer’s instructions. The PCR reaction was conducted as follows: 95 °C for 15 min, followed by 40 cycles of 95 °C for 10 s and 60 °C for 32 s. Sequences of the primers used in RT-qPCR assays are listed in Supplementary Table S2. The relative expression of the target genes was calculated via the 2^−ΔΔ^Ct method. All of the PCR reactions were performed at least three times.

### 3.10. Determination of Total Carbohydrate Content

For the determination of the total carbohydrate content, *B. cinerea* mycelia treated with 5.0 μg/mL and 10.0 μg/mL of isoxanthohumol were used as the treatment group, and the mycelia treated with DMSO were used as a blank control. The effect of isoxanthohumol on the carbohydrate content was determined according to the previously described method [2] using a total carbohydrate content assay kit (Solarbio, Beijing, China). In brief, the harvested mycelium was used to extract total carbohydrates, and the standard curve was prepared. The content of carbohydrate was expressed as milligrams per gram on a fresh weight basis.

### 3.11. Determination of Dehydrogenase Activities and CA Content

Dehydrogenase activities and CA content were measured by a previous method with minor modifications [34]. Mycelia were collected using the above methods. The activities of MDH and SDH were measured using an NAD-malate dehydrogenase assay kit (Solarbio, Beijing, China) and a micro succinate dehydrogenase assay kit (Solarbio, Beijing, China), respectively. One unit is the consumption amount of 1 nmoL 2, 6-dichlorophenol indophenol or NADH per minute under the assay conditions. The content of CA was assessed using a citric acid content detection kit (Solarbio, Beijing, China). All operations were accomplished according to the manufacturer’s recommendation, and each experiment was run at least three times.

### 3.12. Determination of ATP Content and ATPase Activity

For the determination of the ATP content and ATPase activity, mitochondria were isolated using a yeast mitochondria isolation kit (Biovision, San Francisco, CA, USA), and the content of protein was determined using a BCA protein assay kit (Solarbio, Beijing, China). Then, the ATP content and ATPase activity of the mitochondria were determined using an ATP Colorimetric/Fluorometric assay kit and an ATPase/GTPase activity assay kit, respectively. All operations were performed according to the manufacturer’s instruction, and each experiment was performed at least three times.

### 3.13. Determination of Cell Respiration

The effect of isoxanthohumol on the respiration rate was assessed using a Dissolved Oxygen Meter (JPSJ-606L Dissolved Oxygen Meter; LEICI, Shanghai, China) by the methods of (Yan et al., 2020b). Firstly, *B. cinerea* was cultured for three days to obtain mycelia. Then, different concentrations of isoxanthohumol were added to 40 mL of PBS solution containing 1% glucose, and 200.00 mg of mycelia was suspended in PBS solution. Finally, the dissolved oxygen content was recorded for 24 h. The experiment was performed three times. The respiration rate of *B. cinerea* was calculated by the following formula:

Inhibition rate (%) = [1 − (R_0_ − R_1_)/R_0_] × 100

where R_0_ is the dissolved oxygen content at 0 h, and R_1_ is the dissolved oxygen content at 0.5~24 h.

### 3.14. Cell Membrane Permeability

The cell membrane permeability of *B. cinerea* was evaluated by measuring the relative electrical conductivity. In brief, *B. cinerea* was added to the PD medium and then incubated at 23 ± 2 °C for 3 days to obtain the sample. Then, 400.00 mg of mycelia was suspended in 40 mL of sterile distilled water with 5.0, 10.0, 25.0, or 50.0 μg/mL of isoxanthohumol as the treatment group; at the same time, 0.5% DMSO (*v*/*v*) was used as a blank control. Finally, the conductivity of the suspension was determined using a conductivity detector (HORIBA EC1100, HORIBA Advanced Techno Co., Ltd., Osaka, Japan) (conductivity at 0 h was marked as L0, and that at 0.5–12 h was marked as L1). After the mycelia were boiled for 30 min, the final electric conductivity was measured and marked as L2. The relative permeability rate of mycelia was calculated by the following formula:

Relative electric conductivity (%) = [(L1 − L0)/(L2 − L0)] × 100

### 3.15. Determination of Glycerol Content

The glycerol content in the sample was determined using a glycerol assay kit (Nanjing Jiancheng Bioengineering Institute, Nanjing, China). *B. cinerea* was cultured in PD medium at 23 ± 2 °C for 3 days to obtain mycelia. Then, the extraction and determination of glycerol were performed according to the manufacturer’s instructions, and each experiment was repeated at least three times.

### 3.16. Determination of MDA Content

The content of MDA was examined using a micro malondialdehyde assay kit (Solarbio, Beijing, China). After the sample was collected, all operations were carried out according to the manufacturer’s instruction. Each experiment was performed in three replicates.

### 3.17. Determination of H_2_O_2_ Content

The hydrogen peroxide content was measured using a micro hydrogen peroxide assay kit (Solarbio, Beijing, China). All operations were carried out following the manufacturer’s instructions. The sample was collected as described above, and each experiment was repeated at least three times.

### 3.18. Activity of Antioxidant-Related Enzymes

The activity of antioxidant-related enzymes, including SOD, CAT, POD, and APX, was measured using different commercial kits (BC0175, BC0205, BC0095, BC0225, respectively; Solarbio, Beijing, China) [26]. After the extraction, the suspension was centrifuged, and the activity was measured according to the manufacturer’s instructions.

### 3.19. Statistical Analysis

The data were reported as mean ± standard deviations, and significant differences between mean values were evaluated via Tukey’s multiple range test, followed by one-way ANOVA (* *p* < 0.05, ** *p* < 0.01, *** *p* < 0.001). All the statistical analyses were carried out using SPSS (IBM, Amonk, NY, USA) software version 24.0.

## 4. Conclusions

In conclusion, the results of this study show that the ethanolic extract of *H. lupulus*, a medicinal and edible plant, exhibited moderate antifungal activity against the five tested phytopathogenic fungi in vitro. Isoxanthohumol was found to possess strong antifungal activity against *B. cinerea* with an EC_50_ value of 4.32 µg/mL; therefore, the antifungal mechanism of isoxanthohumol on *B. cinerea* was studied systematically. The present data suggest that the antifungal mechanism of isoxanthohumol is mainly related to metabolism; it affected the carbohydrate metabolic process, destroyed the TCA cycle, and hindered the generation of ATP by inhibiting respiration. In addition, isoxanthohumol also affected the membrane system, induced by membrane lipid peroxidation, thus accelerating the death of *B. cinerea* mycelia. This study demonstrates that isoxanthohumol can be used as a potential botanical fungicide for the management of phytopathogenic fungi. To our knowledge, this work is the first report on the antifungal mechanism of isoxanthohumol against *B. cinerea*.

## Figures and Tables

**Table 1:** Antifungal activity of ethanolic extract from *H. lupulus* and isoxanthohumol in vitro.

Compound	Concentration (μg/mL)	Inhibition Rate ^a^ (%) ± SD				
		*R. solani*	*B. cinerea*	*F. graminearum*	*S. sclerotiorum*	*M. oryzae*
Ethanolic extract	500	37.01 ± 0.43	44.42 ± 0.27	48.19 ± 0.54	51.52 ± 0.53	43.40 ± 0.23
	250	25.66 ± 0.18	40.76 ± 0.17	39.56 ± 0.18	47.97 ± 0.47	29.34 ± 0.29
Isoxanthohumol	50	31.90 ± 1.08	85.90 ± 0.24	63.88 ± 0.23	84.46 ± 0.30	33.50 ± 0.27
	25	18.02 ± 0.31	78.45 ± 0.32	60.43 ± 0.82	79.49 ± 0.05	28.10 ± 0.22

^a^ Each treatment repeated three times. Data are displayed as mean ± SD.

**Table 2:** Inhibition of the mycelia of three plant pathogenic fungi by isoxanthohumol.

Pathogenic Fungus	EC_50_ (µg/mL)	Pathogenic Fungus (y = ax + b)	Correlation Coefficient (R^2^)
*S. sclerotiorum*	14.52	y = 3.78x − 4.43	0.973
*B. cinerea*	4.32	y = 2.08x − 1.38	0.836
*F. graminearum*	16.50	y = 1.64x − 2.00	0.976

**Table 3:** The antifungal activity of isoxanthohumol and boscalid in vivo.

Compound	Concentration (μg/mL)	Protective Effect		Curative Effect	
		Lesion Length ^a^ (mm ± SD)	Control Efficacy (%)	Lesion Length (mm ± SD)	Control Efficacy (%)
Isoxanthohumol	50	23.07 ± 2.62	32.35	24.77 ± 1.23	27.39
	100	21.04 ± 3.98	38.32	22.09 ± 0.78	35.23
	200	15.40 ± 2.96	54.84	20.72 ± 1.92	39.25
Boscalid	50	15.16 ± 5.02	42.93	30.78 ± 2.52	9.77
	100	13.53 ± 5.62	49.09	16.90 ± 3.11	50.47
	200	7.98 ± 2.40	69.95	16.71 ± 3.18	51.00
Control	-	26.57 ± 5.04	-	34.11 ± 2.32	-

^a^ Each treatment repeated five times.

## Data Availability

All data are contained within this manuscript.

## Supplementary Materials

The following are available online at https://www.mdpi.com/article/10.3390/ijms221910853/s1. Table S1: Key DEGs involved in Carbon metabolism and TCA cycle and their primer sequences. Table S2: Inhibition of mycelium of three plant pathogenic fungi by isoxanthohumol.
