# Supplementary material for: The Antifungal Mechanism of Isoxanthohumol from Humulus lupulus Linn
Source: Int J Mol Sci. 2021 Oct 7;22(19):10853. doi: 10.3390/ijms221910853 (PMC8509189; doi:10.3390/ijms221910853)
Supplement: Supplementary file 1 [file ijms-22-10853-s001.zip › ijms-1381891-supplementary.pdf]

Supplemental Table S1. Inhibition of mycelium of three plant pathogenic fungi by isoxanthohumol.

**Compound**	**Concentration (μg/mL)**	**Inhibition rate [a] (%) ± SD**		
		Sclerotinia scleotiorum	***Botrytis cinerea***	***Fusarium graminearum***
**Isoxanthohumol**	50	84.46±0.30	85.90±0.24	63.88±0.23
	25	79.49±0.05	78.45±0.32	60.43±0.82
	10	33.28±0.64	61.03±0.43	45.11±0.23
	5	11.15±1.82	57.00±0.60	27.08±1.24
	2.5	5.73±0.14	39.03±0.93	0.00±0.00
	**EC_{50}**	**14.52**	**4.32**	**16.50**

[a] Repeat each treatment three times. Data are displayed as mean ± SD.

Supplemental Table S2. Key DEGs involved in Carbon metabolism and TCA cycle and their primer sequences.

**Gene alias**	**Control _FPKM**	**Treat _FPKM**	**log2 Fold Change**	**Annotation**	**Primer sequences**
BCIN_07g05430	0.273	37.186	6.58	Carbon metabolism	F:CGAGAAGGTGCTGAGTTAGATG<br>R:CCTTTCAGGTTCAGGGAAGATT
BCIN_15g02910	0.790	4.343	2.46	Carbon metabolism	F:CTGTATCCACCCAAGCCTATAC<br>R:TTTCTCTCGTCGTAGCATTCC
Bcboa1	1.436	5.503	1.94	Carbon metabolism	F:CAATCACTGACGAACCCTCTAA<br>R:AACCAGTCTCCATGCTCTTTC
Bcpio2	0.393	1.596	1.81	Carbon metabolism	F:ATGCCACCAAGACCATCAC<br>R:CATCTCCGTCGCGTCAATAA
BCIN_12g00360	137.170	51.030	-1.33	Carbon metabolism	F:CTCGTCTGTATGGCCGTATTT<br>R:CAAACCGACGAAGCACATTAC
BCIN_02g02750	316.836	529.293	0.82	Citrate cycle (TCA cycle)	F:GGATTTGGCGAGAACAAGGA<br>R:GTGAGTGGTGTGAGCAGAAA

BCIN_05g04430	176.703	288.606	0.79	Citrate cycle (TCA cycle)	F:TTGAATCCAGCTACCGATGC R:GCACTCTCTTCGATGGGAAATA
